# A low‐coverage skim‐sequencing and imputation pipeline for genomic selection

**DOI:** 10.1002/tpg2.70139

**Published:** 2025-10-23

**Authors:** Sajal R. Sthapit, Jared Crain, Steve Larson, James A. Anderson, Prabin Bajgain, Lee R. DeHaan, Jesse Poland

**Affiliations:** ^1^ The Land Institute Salina Kansas USA; ^2^ Department of Plant Pathology Kansas State University Manhattan Kansas USA; ^3^ USDA‐ARS Forage and Range Research Lab Logan Utah USA; ^4^ Department of Agronomy and Plant Genetics University of Minnesota Saint Paul Minnesota USA; ^5^ USDA‐ARS United States Dairy Forage Research Center Madison Wisconsin USA; ^6^ Center for Desert Agriculture King Abdullah University of Science and Technology Thuwal Saudi Arabia

## Abstract

Genomic selection (GS) can accelerate plant breeding gains by reducing breeding cycle times, reducing phenotyping costs, or improving selection accuracy. GS is especially promising for perennial crops such as intermediate wheatgrass (IWG, *Thinopyrum intermedium*) that may require multiple years of evaluation under phenotypic recurrent selection. A major obstacle in implementing GS is the need for an affordable, high‐density, genetic marker system that is scalable to thousands or tens‐of‐thousands of samples in breeding programs, especially in emerging or minor crop species. As sequencing costs continue to decrease, low‐coverage whole genome skim‐sequencing (skim‐seq) has become an attractive method for GS. Using commercial laboratory products and open‐source software, we implemented whole genome prediction at breeding program scale using ultra‐low coverage (0.01x– 0.05x, 100–125 million reads per sample) whole genome skim‐seq. Using STITCH (Sequencing to Imputation Through Constructing Haplotypes) imputation software, we evaluated optimization of imputation parameters including sequence coverage and number of assumed ancestral haplotypes. Finally, we evaluated whole genome prediction cross‐validation accuracies using reduced representation genotyping‐by‐sequencing (GBS) versus skim‐seq data for IWG, an outcrossing, heterozygous, large genome (12.7 Gb), polyploid perennial species. Our results indicate correlations between cross‐validation accuracies across five traits in IWG using skim‐seq data (*r* = 0.29–0.61) can be used as effectively as GBS (*r* = 0.29–0.55) while generating low‐coverage archival sequence data that will be robust to technological advances. These methods will be applicable to a wide range of crops and scale to breeding program size, allowing for more tractable implementation of GS within breeding programs.

## Core Ideas

- Skim‐sequencing, ultra‐low 0.05x genome coverage, was used to implement genomic prediction.
- Equivalent cross‐validation genomic prediction accuracies were achieved by skim‐seq and genotyping‐by‐sequencing.
- A bioinformatics pipeline from single nucleotide polymorphism discovery to imputation was developed, scalable to tens of thousands of samples.
- Skim‐seq leverages technological advances in sequencing, creating robust data resources for future applications.
- Skim‐seq alleviates barriers to entry for genomics‐assisted breeding in low‐resourced species.

## Plain Language Summary

Plant breeding has been revolutionized by advances in genotyping technology. With the ability to genetically profile large populations, plant breeders can more effectively select plants that have greater agronomic performance based completely on their genetic makeup. However, few plant breeding programs have adopted these technological advances due to the challenges and cost associated with genotyping plants at a breeding scale (thousands) of plants. We investigated low‐cost sequencing techniques and computational methods to infer each plant's genetic makeup. Our results indicate that these methods can be used effectively, providing a road map for low‐resourced species and breeding programs to exploit the genomics revolution through open‐source, non‐proprietary methods. Furthermore, the data generated can be used for additional genetic studies.

**Abbreviations**
BLUP: best linear unbiased prediction
GBS: genotyping‐by‐sequencing
GEBV: genomic estimated breeding value
GS: genomic selection
IQS: imputation quality score
IWG: intermediate wheatgrass
MAF: minor allele frequency
STITCH: Sequencing to Imputation Through Constructing Haplotypes
TLI: The Land Institute
UMN: University of Minnesota
WGS: whole‐genome sequencing

## 1 INTRODUCTION

Genomic selection (GS) uses a training population of genotyped and phenotyped individuals to create a model to predict genomic estimated breeding values (GEBVs) for selection candidates based solely on their genotypes (Meuwissen et al., 2001). As a result, breeders can genotype selection candidates, predict their GEBVs, and select the best individuals even before phenotypic evaluation occurs. The ability to make selection at the seedling stage or earlier in the breeding program accelerates the breeding process and allows for faster genetic gains. In cases where phenotyping adult plants is more expensive than genotyping, larger populations can be evaluated, and hence greater selection intensity can be applied (Hickey et al., 2017).

A genotyping platform suitable for GS needs to be (i) low‐cost, (ii) high‐throughput for use on large breeding populations, and (iii) cover the entire genome with markers (Wartha & Lorenz, 2021). These criteria enable genome‐wide capture of linkage disequilibrium and optimize resource utilization when implemented in large populations where selection results in discarding a large proportion of genotyped individuals. Since GS was proposed (Meuwissen et al., 2001), several markers systems have been used to implement GS with most markers platforms being either array‐based or sequence‐based (Elbasyoni et al., 2018; Poland, Endelman, et al., 2012).

Genotyping on single nucleotide polymorphism (SNP) arrays generates high‐confidence genotype calls with a low level of missing data (Burridge et al., 2024; Hong et al., 2012) but can be costly to design. The SNP calling itself is directly related to the germplasm panel used to design the array, potentially creating ascertainment bias (Burridge et al., 2024). Genotyping‐by‐sequencing (GBS) is another genotyping method that is used for GS without the need for SNP arrays or a reference genome (Poland, Endelman, et al., 2012). GBS, and related methods, is a reduced‐representation approach that generates DNA libraries by digesting DNA at specific cut‐sites using restriction enzymes (Elshire et al., 2011; Poland, Brown, et al., 2012). A strength of GBS is that it relies on the genotyped individuals for SNP calls, limiting the ascertainment bias common in array development (Heslot et al., 2013; Poland & Rife, 2012). It produces high‐confidence genotype calls but has a higher rate of missing calls than SNP arrays when sequenced at lower coverage that is commonly used for most applications. In comparison to SNP arrays that typically have less than 10% missing data, published GBS applications routinely use SNP loci that have up to 80% of the individual loci missing SNP calls (Crain, Haghighattalab, et al., 2021; Poland, Endelman, et al., 2012). A limitation of GBS is that marker sets can vary substantially between populations and have potential licensing issues (Bernardo et al., 2020).

While the cost of whole‐genome sequencing (WGS) continues to decrease, it is still costly to apply to large breeding populations, especially for species with large genomes like intermediate wheatgrass (IWG, *Thinopyrum intermedium*). Intermediate wheatgrass is a close relative of annual wheat and is an outcrossing, allohexaploid with a large genome estimated to be 12.75 Gb (Vogel et al., 1999). The IWG genome is comprised of 21 chromosomes divided into three subsets of seven chromosomes, where each set is homologous to barley (Kantarski et al., 2016). The sets of chromosomes (subgenomes) are labeled 1J‐7J, 1S‐7S, and 1V‐7V, where the naming is based on homologies to possible diploid ancestors of IWG (Crain et al., 2022). While IWG is a hexaploid and the genome constitution is actively debated (Wang et al., 2025), the chromosome sets have been designated as diploid genomes (Q. Chen et al., 1998).

WGS is attractive as the data it generates provides flexibility for use and reuse in various studies over the long term. Even low (0.5x–1.0x) coverage WGS that divides the available sequencing effort across a large number of samples is sufficient and often better than SNP arrays for a variety of population genomics studies (Fumagalli, 2013; Lou et al., 2021). Ultra‐low (0.01x–0.5x) coverage whole genome skim‐sequencing (skim‐seq) protocols using off‐the‐shelf library preparation products have now been used in wheat and non‐model species like IWG (Adhikari et al., 2022). Thus, there is the potential of utilizing skim‐seq for GS due to the combined value of being a low‐cost and high‐throughput platform.

Utilization of missing data, either from arrays or sequence‐based data, for genetic studies has relied upon imputation. Often, imputation has been used as a strategy to reduce the long‐term cost of using a high‐density SNP array. To achieve lower genotyping cost, typically, a reference panel is prepared by genotyping a diverse population with a dense SNP array or high‐coverage whole genome sequencing. Then, a sparse SNP array, which has a small subset of the loci in the reference panel, is used to genotype the study population. Finally, an imputation algorithm matches the haplotype segments from the study population to a reference haplotype using the common loci to infer genotypes at the untyped loci (Marchini & Howie, 2010). Within human genomics, large reference panels including 133,597 human genomes are available for use (NHLBI, 2025; Taliun et al., 2021), and version 5 of the popular imputation software Beagle can now efficiently use reference panels with millions of samples (Browning et al., 2018). However, SNP arrays and adequate reference panels are expensive to develop, especially in highly diverse species with large genomes.

Imputation based on low‐coverage skim‐seq data has been facilitated by development of the imputation software package STITCH (Sequencing to Imputation Through Constructing Haplotypes), which was designed to use low‐coverage sequence reads rather than high‐confidence genotypes without the need for a reference panel (Davies et al., 2016). Although STITCH requires a reference genome for read alignments, it leverages large sample sizes that are typical of a plant breeding program to mitigate the need for extensive reference panel development. Additionally, it has been tested in outbred, heterozygous populations (Davies et al., 2016) sharing characteristics of IWG. With the goal of effectively implementing skim‐seq for GS, our objectives were to (1) use open‐source tools, including STITCH, to assemble a pipeline to process and impute skim‐seq reads as a viable marker set for GS; (2) evaluate the accuracy of skim‐seq imputed genotypes; and (3) assess the effectiveness of GS using skim‐seq genotypes compared to more commonly used GBS data.

**Core Ideas**

- Skim‐sequencing, ultra‐low 0.05x genome coverage, was used to implement genomic prediction.
- Equivalent cross‐validation genomic prediction accuracies were achieved by skim‐seq and genotyping‐by‐sequencing.
- A bioinformatics pipeline from single nucleotide polymorphism discovery to imputation was developed, scalable to tens of thousands of samples.
- Skim‐seq leverages technological advances in sequencing, creating robust data resources for future applications.
- Skim‐seq alleviates barriers to entry for genomics‐assisted breeding in low‐resourced species.

## 2 MATERIALS AND METHODS

### 2.1 Plant materials

Breeding populations included the IWG breeding programs at The Land Institute (TLI, Salina, KS), the Forage and Range Research Agriculture Research Service unit (FRR, Logan, UT), and the University of Minnesota (UMN, Minnesota). The breeding programs at each location have been described in detail by Bajgain et al. (2023). The current IWG domestication and breeding efforts in the United States started at the Rodale Institute in the 1980s from an evaluation of 250 accessions (Crain et al., 2024; Wagoner, 1990). In the first breeding cycle at the Big Flats Plant Materials Center in Corning, NY, 14 selected genets were used to establish subsequent generations. As IWG is primarily outcrossed and heterozygous, we utilize the term “genet” to refer to a genetically unique individual and “genotype” to refer to the exact DNA sequence of an individual (Zhang et al., 2016). Genet is often used by ecologists to describe an organism that is genetically unique (Beeby & Brennan, 2008) and is more appropriate than calling IWG a “line” or “variety” as each plant has its own genetic constitution (Zhang et al., 2016). The TLI breeding program was developed almost exclusively from progeny of this first group of selected genets, which would indicate a maximum of 28 founder haplotypes in the current program (Crain et al., 2024; Zhang et al., 2016). Likewise, the FRR and UMN breeding programs began with material that primarily traces back to TLI (Bajgain et al., 2023). The FRR, TLI, and UMN programs utilize GS for breeding, creating a sizeable number of samples that have been genotyped. Plant material used for imputation include 4895 genets from the TLI program spanning six generations and 4885 genets from the FRR program covering two generations (Table 1). A total of 51 genets from the UMN breeding material were also included in the samples used for variant discovery. For cross‐validation of GS predictions, we used a subset of 4226 genets from TLI‐Cycles 10–12 that were genotyped with GBS and phenotyped for multiple traits including percent free‐threshing seed, plant height, seed mass, shattering, and spike yield (Table 1).

**TABLE 1:** Number and source of IWG genets used to develop the skim‐seq imputation pipeline.

Breeding program	Germplasm generation	Imputation (∼0.05x)	Variant discovery (∼2x)	Accuracy evaluation (∼17x)	GBS and phenotyping
TLI	Cycle 4	–	2	2	–
TLI	Cycle 6	–	1	1	–
TLI	Cycle 7	112	100	9	–
TLI	Cycle 8	95	93	–	–
TLI	Cycle 9	98	27	–	–
TLI	Cycle 10	1115	141	24	992
TLI	Cycle 11	1836	10	10	1724
TLI	Cycle 12	1639	–	–	1510
TLI	Sub‐total	4895	374	46	4226
FRR	Training	2224	20	–	–
FRR	Selection	2661	–	–	–
FRR	Sub‐total	4885	20	–	–
UMN	Parents	–	51	–	–
All	Grand total	9780	445	46	4226

Abbreviations: GBS, genotyping‐by‐sequencing; IWG, intermediate wheatgrass; FRR, Forage and Range Research unit; skim‐seq, skim‐sequencing; TLI, The Land Institute; UMN, University of Minnesota.

### 2.2 DNA extraction

Approximately 50 mg of fresh leaf tissue was collected from IWG seedlings and placed in 96‐well plates. Most of the tissue collection was in conjunction with the active IWG breeding program, with DNA extracted during each cycle and subsequently aliquoted for skim‐seq libraries and GBS. A blank well was left randomly in each plate as a negative control. DNA for all samples was extracted with the MagMAX Plant DNA Isolation kit (ThermoFisher Scientific) following the manufacturer's instructions. The same DNA samples were used for both skim‐seq and GBS libraries.

### 2.3 Genomic sequencing

#### 2.3.1 Skim‐sequencing

Ultra‐low coverage whole genome sequencing libraries were prepared according to a modified protocol presented by Adhikari et al. (2022). Multiplexing of genomic libraries varied from 576 to 1248 samples (6 to 13 plates) per library (Adhikari et al., 2022). Each library was sequenced on single or multiple flowcell lanes using the Illumina NovaSeq and NovaSeqX+ platforms to obtain a target coverage of 0.05x for each sample. Genets that had been used as parents within the breeding program were sequenced to a higher coverage of 0.2x–2x, where deeper sequencing was achieved by reducing the level of multiplexing per library and sequencing on multiple lanes. For testing an ultra‐low‐coverage skim‐sequencing and imputation pipeline, samples with coverage of greater than 0.2x were down sampled to 0.05x coverage using seqtk (https://github.com/lh3/seqtk). All skim‐seq libraries were sequenced at Psomagen Inc.

#### 2.3.2 High‐coverage sequencing

Complementing the low‐coverage sample data, we sequenced 46 genets from the TLI breeding program (Table 1) at higher coverage (∼17x) using Illumina TruSeq DNA PCR‐free preparation. Genomic DNA was sheared to obtain libraries with a 350 bp insert size. The high‐coverage samples were to obtain high‐confidence genotype calls that were used to evaluate imputation accuracy.

#### 2.3.3 Genotyping‐by‐sequencing

For the 4226 genets that had both skim‐seq and GBS libraries (Table 1), DNA was originally extracted for the breeding program and aliquoted for subsequent skim‐seq libraries. For IWG, we followed the same procedure as outlined by Crain, DeHaan, et al. (2021) and Crain, Haghighattalab, et al. (2021), and highlight the protocol here. The GBS protocol used for library preparation included a two‐enzyme digestion (Poland, Brown, et al., 2012) and multiplexing of 192 samples per library. Libraries were sequenced in conjunction with the breeding program from 2016 to 2022 using Illumina sequencing machines (HiSeq 2500, HiSeqX, NextSeq 500) and multiple service vendors including Hudson Alpha (Huntsville, AL), Psomagen Inc. (Rockville, MD), and Kansas State University Integrated Genomics Facility (Manhattan, KS). Following library sequencing, the TASSEL GBSv2 pipeline (Glaubitz et al., 2014) and version 3.1 of the IWG draft reference genome (https://phytozome‐next.jgi.doe.gov/info/Tintermedium_v3_1) were used to call variants using only uniquely aligning tags with a minimum of 50 bp length. Homozygous calls required a read‐depth greater than four, while heterozygous calls were made with sequencing depth of two contrasting tags. After genotype calling, data were filtered to remove loci with missing data greater than 70% and loci with a minor allele frequency (MAF) less than 1%. IWG samples were removed if they had more than 95% missing data. The 4654 IWG samples and 31,393 loci passing all filters were imputed using Beagle version 4.1 (Browning & Browning, 2016) without a reference panel using default parameters. Only 4226 samples from Cycles 10–12 of the TLI breeding program with phenotyping data for plant height, seed mass, shattering, and spike yield as described by Crain, DeHaan, et al. (2021) were included for GS cross‐validation.

### 2.4 Whole‐genome sequencing bioinformatic processing

#### 2.4.1 Sequence files to BAM files

Sample processing for whole genome sequencing data (skim‐seq and high‐coverage samples) included generating the demultiplexed fastq files using Illumina's BCL software. The fastq files for each genet were then processed through a standard pipeline. Adapters were trimmed with fastp (S. Chen et al., 2018). Reads with a minimum 75 bp length were aligned to the IWG draft reference genome (https://phytozome‐next.jgi.doe.gov/info/Tintermedium_v3_1) using HISAT2 (Kim et al., 2019) to create sequence alignment map (SAM) files. Within HISAT2, the maximum fragment length for valid paired‐end alignment was set to 1000 bp compared to the default of 500 bp (‐X 1000). Samtools (Danecek et al., 2021) was used to filter for unique, concordant alignments with quality score greater than 60, followed by sorting and writing indexed BAM (binary version of SAM) files to use in the imputation pipeline.

#### 2.4.2 Variant ascertainment

To ascertain high‐quality variants, we first called variants on 445 genets (Table 1) from the TLI, FFR, and the UMN IWG breeding programs (Bajgain et al., 2023; Crain, DeHaan, et al., 2021). The BCFtools “mpileup” and “call” commands were used to call variants to the IWG reference genome (Danecek et al., 2021). Next, we filtered for variants that were biallelic with a minimum quality score of 60, read‐depth within two standard deviations from the mean population sequencing depth, calls present in at least half of the 445 samples, and with a MAF > 5%. We considered variants that passed all these filters as high‐quality variants, and they were used to create chromosome‐specific position files to use as input for STITCH. Approximately 1 million high‐quality variants were included for imputation per chromosome (28.2 million total genome variants).

#### 2.4.3 Imputation using STITCH

**FIGURE 1:**
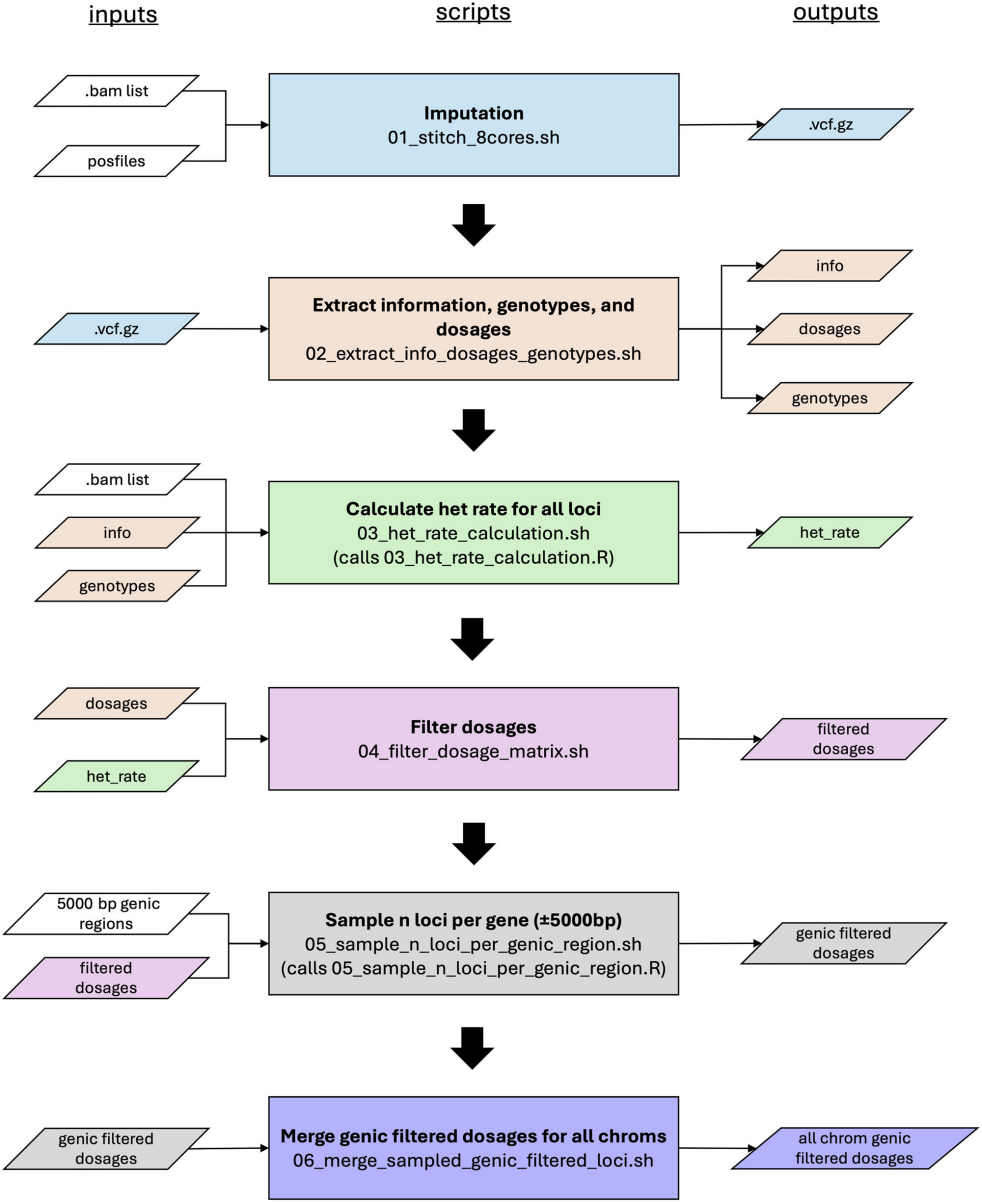
Pipeline for imputation and post‐processing of imputed variant call format (VCF) file to create filtered dosage matrix for use in genomic prediction. Color of the outputs match the script used to generate it.

We used STITCH v. 1.6.9 (Davies et al., 2016) for imputation in our bioinformatics pipeline (Figure 1). Davies et al. (2016) recommend testing imputation using different numbers of ancestral haplotypes (*K*) to identify the appropriate value of *K* for the population being studied. We evaluated imputation on all chromosomes at seven different levels of *K* (4, 8, 12, 16, 20, 24, and 28) with 28 representing an upper boundary based on known population demographics. As imputation with higher number of *K* can significantly increase computation time, we worked to identify a large enough *K* to not compromise imputation accuracy with sufficiently fast computation time. For all evaluations, we used 15 for number of generations based on TLI breeding cycles (nGen = 15), although STITCH is robust to misspecification of the nGen parameter (Davies et al., 2016). To speed imputation, each chromosome was imputed as a separate array job with 8 cores and 24 GB memory per core per chromosome (192 GB total memory).

#### 2.4.4 Post‐imputation processing

The compressed variant call format (VCF) output from STITCH was processed and filtered to generate a dosage matrix and a genotype call matrix (Figure 1). Dosage in the STITCH output VCF file represents the expected number of alternate alleles, which can range from zero (homozygous reference, i.e., no alternate allele) to two (two copies of the alternate allele) (Davies, 2021). Genotype calls are reported by STITCH when the posterior probability of the genotype exceeds 90% (Davies, 2021). The genotype calls in the VCF file were used to calculate the heterogeneity rate for all loci. To obtain a final and smaller set for GS, all imputed loci were filtered for STITCH info score >0.80, heterogeneity rate between 5% and 50%, and MAF > 1%. We used a MAF greater than 1% based on the large sample size (*n* = 9780) and to match parameters used within the breeding program (Crain, DeHaan, et al., 2021). To further reduce the dataset size and be more representative of numbers of markers used in GBS, we drew random samples of 32,000, 64,000, and 96,000 variants, as well as random samples of one (36,071 markers), two (64,001 markers), and three variants (88,525 markers) from a region within 5000 bp upstream and downstream of known IWG gene models (genic regions). All skim‐seq datasets for genomic prediction were filtered from the entire sample of 9780 genets, which were imputed together to leverage greater amounts of sequence data.

### 2.5 Accuracy evaluation

#### 2.5.1 Validation set genotypes

For the 46 high‐coverage genets, BCFtools “mpileup” and “call” commands were used to call genotypes of each sample. The validation set (46 high‐coverage genets) was filtered for variants with minimum quality score of 60, a maximum of two alleles, and a read‐depth within ±2 standard deviations (read‐depth = 228–1355) of the mean total coverage of these 46 samples. We did not apply any MAF filter for the validation set loci.

#### 2.5.2 Accuracy evaluations

The raw fastq files of the 46 high‐coverage validation genets were down sampled using seqtk (https://github.com/lh3/seqtk) to four different levels of sequencing coverage: 0.01x, 0.04x, 0.07x, and 0.10x. Imputations were run on these 184 down sampled files along with the 9780 genets from the FRR and TLI breeding populations. Imputation accuracy was evaluated using three metrics: concordance, imputation quality score (IQS), and *R* ^2^. Concordance was defined as the exact match between the known validation set genotype and the imputed genotype. IQS is concordance that has been adjusted for allele frequency (Lin et al., 2010). Finally, *R* ^2^ is the squared correlation between the alternate allele count (0, 1, or 2) in the validation set genotype and the alternate allele dosage (continuous from 0 to 2) in the imputed genotype. Only high confidence genotype calls (minimum depth = 6 and maximum depth = 34 per sample) from the validation set were used to calculate imputation accuracy.

### 2.6 Comparison of genomic predictions

We paired the genomic data with phenotype data that were collected in the TLI breeding program for free‐threshing seed, plant height, seed mass, seed shattering, and spike yield. The phenotypic data represented four calendar years (2021–2024) and three cycles of information measured on 4263 IWG genets. The phenotypic data were on spaced plants as described in Crain, DeHaan, et al. (2021) and Crain, Haghighattalab, et al. (2021), only in advanced generations in the breeding program. Best linear unbiased predictions (BLUPs) were made for each IWG sample using a linear mixed model that has been used in the IWG breeding program that accounts for spatial location, genetic relatedness, and multiple sampling timepoints across years (Crain et al., 2022). The mixed model was implemented with ASREML version 4.1 (Gilmour et al., 2015). Using the same observed phenotypes, BLUPs were generated on the five traits using seven different genotypic datasets (GBS, one marker per gene space, two markers per gene space, three markers per gene space, 32,000, 64,000, and 96,000 random skim‐seq datasets). We evaluated pairwise correlation between the BLUPs generated where only the genotyping dataset differed between models.

To further test the BLUPs for prediction in GS, a fivefold cross‐validation was run by randomly assigning the samples to five different groups. For each group, 80% of samples were used as a training population to predict the GEBVs of BLUPs for the remaining 20%. This step was repeated five times until all samples had been predicted. The correlation between the masked BLUP and the averaged field data across multiple years was used to assess the impact of different genotyping datasets for genomic prediction. Averaged data were used to avoid including the same marker matrix for both the phenotypic field evaluation and the GS model development (Crain, Haghighattalab, et al., 2021). A 95% confidence interval was constructed around the correlation value; correlation values that did not fall within the confidence interval were declared statistically different.

The R package *rrBLUP* (Endelman, 2011) was used to develop genomic relationship matrices and genomic predictions. Shell scripts for STITCH imputation, post‐processing, and analysis were run on the Kansas State University's Linux‐based Beocat Computing Cluster. R scripts for GS and evaluation were run on a Windows personal computer.

## 3 RESULTS

### 3.1 Variant ascertainment

The 445 genets used for variant discovery had a mean sequencing coverage of 1.79x (i.e., a total population read‐depth of 797x) and standard deviation of 0.65x. After applying filters for read‐depth of 218–1375 (mean ± 2 standard deviations) and other filtering metrics, we identified 28.2 million genome‐wide high‐quality SNP variants (Table S1). We observed variation across the sub‐genomes, where the J genome had an average of 1.7 million variants per chromosome and the S and V genomes had averages of 1.1 million and 1.2 million variants per chromosome, respectively. Likewise, the variant density differed across the subgenomes ranged from one SNP per 364 bp (J genome) to one SNP per 493 bp (V genome). The S genome had an average of one SNP per 412 bp. The number of variants ascertained was correlated (*r* = 0.77) with the size of the chromosome (Figure S1). Chromosome 4S had more variants for its length in base pairs (11,217 variants per Mb) compared to the genome average of 8405 variants per Mb for other chromosomes. Additionally, we note that this variation is not exhaustive. In the 46 high‐coverage genets sequenced at ∼17x coverage, we identified 97.3 million biallelic variants with MAF > 5% (Table S1).

**FIGURE 2:**
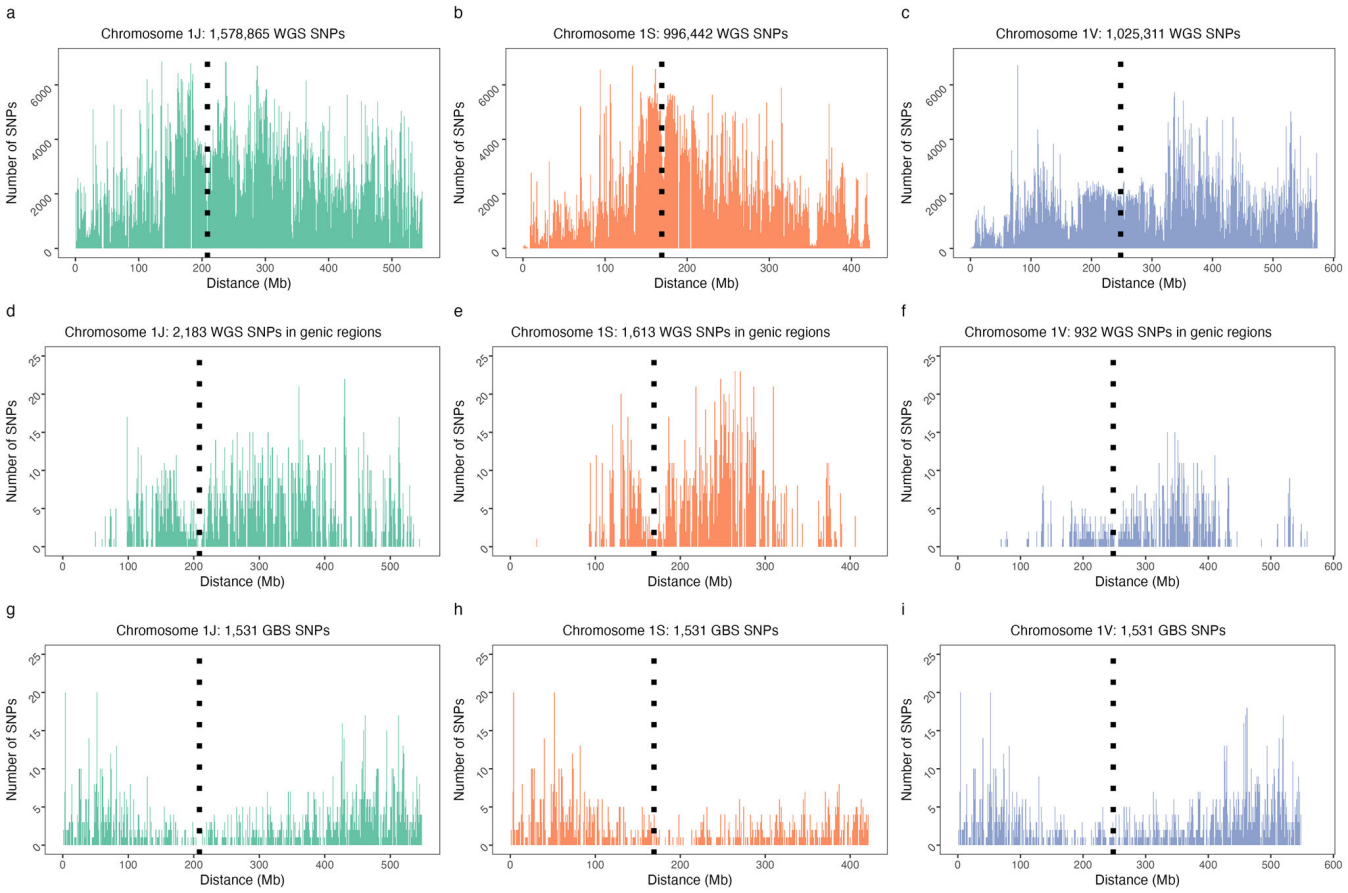
Distribution of number of variants (*y*‐axis) ascertained in chromosomes 1J (a, d, and g), 1S (b, e, and h), and 1V (c, f, and i) along the length of the chromosome (*x*‐axis) represented in 1 Mb bins. Variants were identified by whole‐genome resequencing of 445 intermediate wheatgrass samples at ∼2x coverage (a–c), whole‐genome sequencing single nucleotide polymorphism (SNPs) filtered for info score > 0.80, heterozygosity rate of 5%–50%, minor allele frequency > 1%, and one marker within 5000 bp of a known gene model (d–f) and SNPs from genotyping‐by‐sequencing (g–i). Dotted lines are the locations of the centromeres. GBS, genotyping‐by‐sequencing; WGS, whole‐genome sequencing.

Variants discovered from WGS were distributed across the chromosome length without exhibiting a decrease in the pericentromeric region that is typically found with GBS markers (Figure 2; Figures S2–S4). The GBS libraries were prepared with methylation‐sensitive enzymes which likely result in fewer called sites in centromeric and repetitive regions (Poland & Rife, 2012).

### 3.2 Accuracy of imputation at different info scores

**FIGURE 3:**
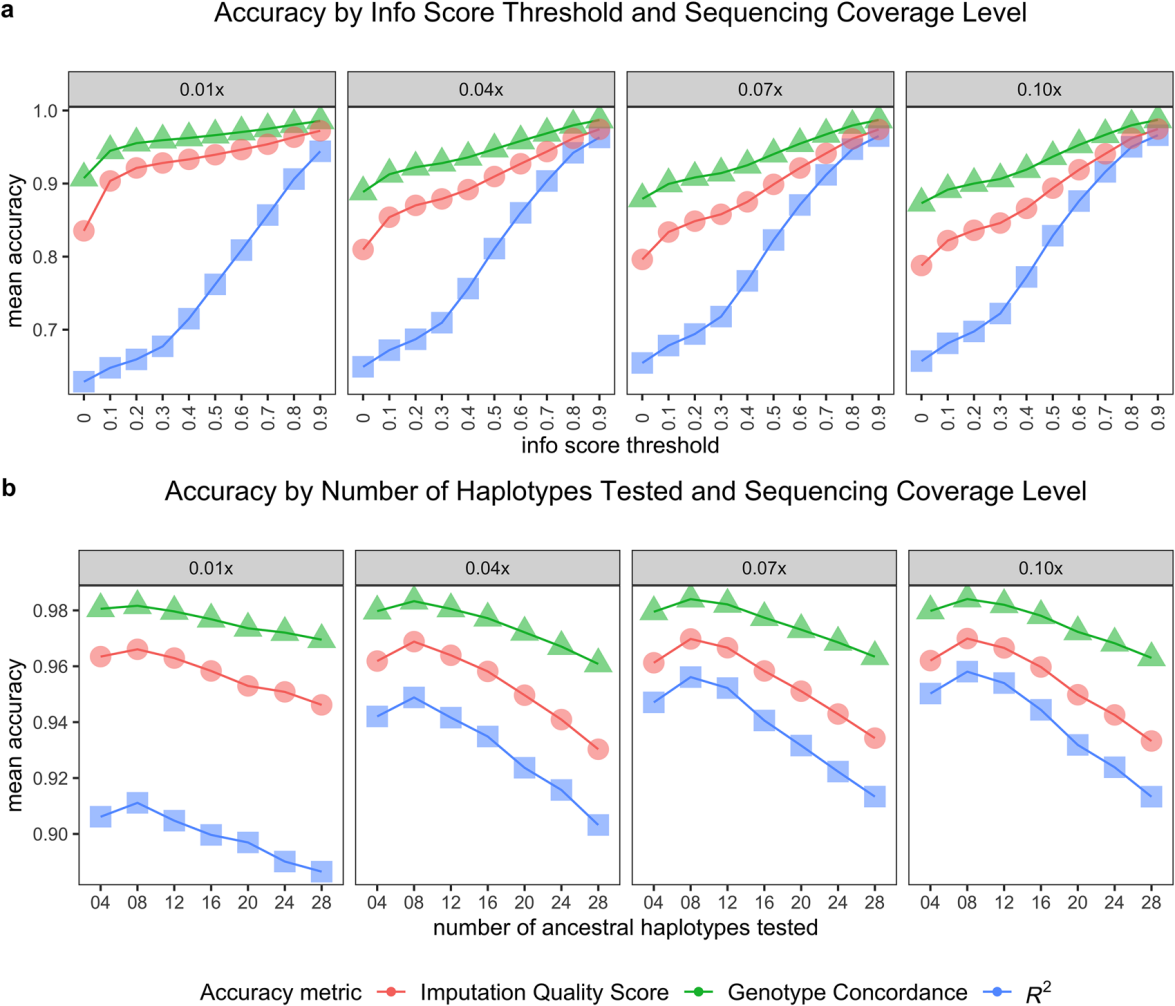
Change in mean accuracy of imputation in chromosome 6V of intermediate wheatgrass at different (a) info score thresholds faceted by sequencing coverage with the number of ancestral haplotypes set to 4 and (b) number of ancestral haplotypes assumed in imputation faceted by sequencing coverage with imputed loci filtered for info score >0.80. The three accuracy metrics represent imputation quality score, genotype concordance, and *R* ^2^ correlation.

We assessed imputation accuracy using genotype concordance and IQS between high‐coverage validation genotypes and imputed genotypes as well as squared correlations (*R* ^2^) between validation and imputed genotype dosages at info score thresholds ranging from 0 to 0.90 in 0.10 increments. Overall, all three metrics of accuracy increased at higher info score thresholds, but the trajectory of increase was different for metrics using imputed genotype calls (concordance and IQS) versus *R* ^2^ (Figure 3a). Even a low threshold of info score >0.10 greatly improved genotype concordance and IQS, but the increase in *R* ^2^ was modest at info score thresholds <0.40. Based on these findings, we developed the bioinformatics pipeline to filter for SNP loci that had info score >0.80. At *K* = 12, 503,556, genic loci (<2% of imputed loci) passed filter levels of info score >0.80, heterozygosity < 0.80, and MAF > 0.01.

At *K* = 8, concordance started above 0.85 at an info score threshold of 0 and kept increasing, with concordance exceeding 0.97 at info score > 0.90 in all chromosomes (Figure S5a). Likewise, IQS started above 0.80 for all chromosomes and exceeded 0.95 at info score > 0.90 (Figure S5b). Squared correlations (*R* ^2^) between validation set genotypes and imputed genotype dosages were above 0.60 at info score > 0 and increased to 0.90 at info score > 0.90 for all chromosomes (Figure S5c).

### 3.3 Effect of the number of ancestral haplotypes

**FIGURE 4:**
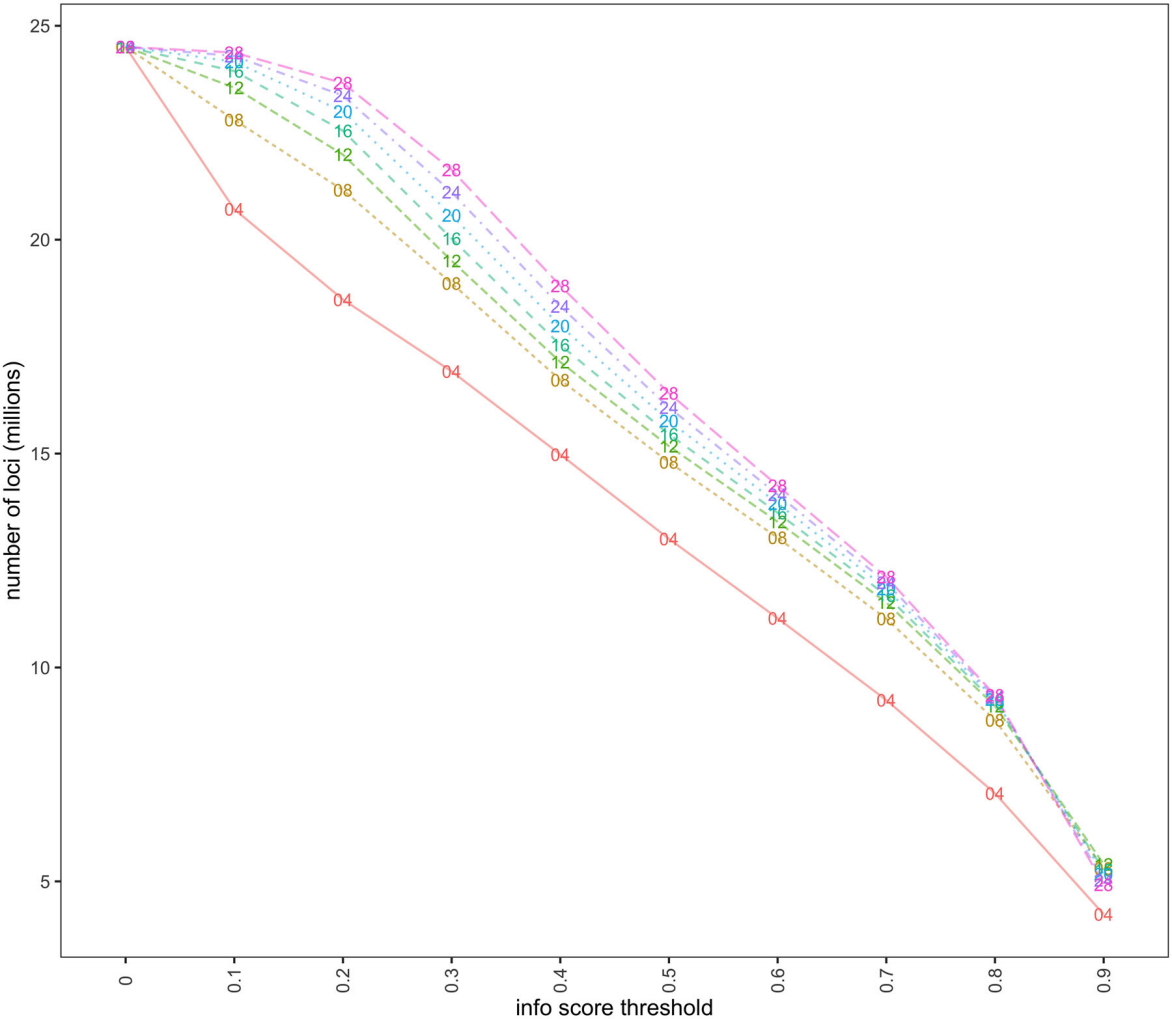
Number of loci (*y*‐axis) exceeding different info score thresholds (*x*‐axis). Points on the line are indicated by the number of ancestral haplotypes modeled in STITCH imputation.

For most chromosomes, the mean accuracy (concordance, IQS, and *R* ^2^) peaked at *K* = 8 or 12 (Figure 3b; Figure S6). At any given info score threshold above 0, imputation at higher values of *K* resulted in more loci passing the pipeline filters. The largest increase in number of loci passing a given quality threshold was highest when going from *K* = 4 to *K* = 8 and more modest at higher values of *K* (Figure 4). We concluded that *K* = 8 or 12 and info score threshold > 0.80 were optimal settings for our IWG population to achieve high accuracy and fast computing time.

### 3.4 Effect of sequencing coverage

**FIGURE 5:**
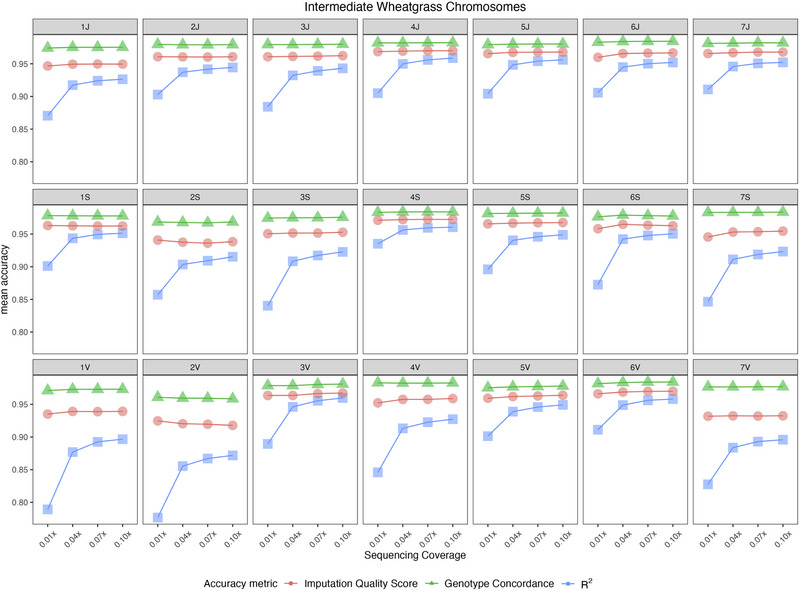
Change in mean accuracy of imputation in intermediate wheatgrass (IWG) chromosomes at different sequencing coverage. Markers in the plot are filtered for info score > 0.80. The three accuracy metrics represent imputation quality score, genotype concordance, and *R* ^2^ correlation.

As samples randomly vary in sequencing coverage around the intended target coverage of 0.05x, we assessed imputation accuracy at varying levels of sequencing depth. Among the three metrics of accuracy, *R* ^2^ benefited the most with an increase in sequencing coverage (Figure 5). At a given info score threshold, *R* ^2^ was lowest at 0.01x coverage and highest at 0.10x coverage. Increase in *R* ^2^ was largest from 0.01x to 0.04x, but more modest for the subsequent increases in coverage, suggesting that 0.05x target coverage was a reasonable goal for breeding scale utilization. The changes in accuracy measured as genotype concordance or IQS at different levels of coverage were small (Figure 5).

### 3.5 Effect on genomic predictions

We developed phenotypic BLUPs for five traits using seven different genotype datasets. These datasets were developed to have approximately the same number up to three times the number of markers as GBS (Table 2) as well as comparing randomly chosen markers compared to markers in defined genic regions. The BLUPs were highly correlated to each other (Figure S7), suggesting that regardless of marker dataset equivalent information was being assessed for field evaluations. Across the fivefold cross‐validation, we observed statistically significant differences between the masked and predicted BLUPs for traits including free‐threshing, seed mass, seed shattering, and spike yield.

**TABLE 2:** Correlations between masked BLUP and predicted GEBV values for five traits using different sets of genotyping data for generating genomic relationship matrices and predictions over fivefold cross‐validations.

Genomic marker set	Loci	Free‐threshing	Plant height	Seed mass	Shattering	Spike yield
Genotyping‐by‐sequencing	30834	0.55ab	0.29a	0.54bc	0.53c	0.41a
Skim‐seq: 1 loci per gene space	36071	0.54bc	0.31a	0.59a	0.61a	0.37b
Skim‐seq: 2 loci per gene space	64001	0.57a	0.31a	0.58ab	0.59ab	0.36bc
Skim‐seq: 3 loci per gene space	88525	0.54bc	0.31a	0.58ab	0.59ab	0.36bc
Skim‐seq: 32,000 random	32000	0.52c	0.29a	0.56abc	0.57b	0.34bc
Skim‐seq: 64,000 random	64000	0.53bc	0.30a	0.56abc	0.57b	0.34bc
Skim‐seq: 96,000 random	96000	0.52c	0.30a	0.56abc	0.58b	0.34bc

*Note*: From a population of 4226 samples, 80% were used as the training population to predict the GEBVs of the remaining 20% with the process repeated five times until all samples were predicted. Both the Beagle‐imputed GBS and STITCH‐imputed skim‐seq data were filtered for heterozygosity rate of 5%–50% and minor allele frequency > 1%. The skim‐seq data were also filtered for info score > 0.80. Trait means that do not share a letter are outside of the 95% confidence interval.

Abbreviations: BLUP, best linear unbiased prediction; GBS, genotyping‐by‐sequencing; GEBV, genomic estimated breeding value; skim‐seq, skim‐sequencing.

Importantly for plant breeding, when considering the truncation selection ranking of the bottom 100 or top 100 individuals (Figures S8 and S9), it was very similar among skim‐seq dataset with loci within genic regions (76%–91% overlap of genets) and among skim‐seq datasets with random loci distribution across the genome (75%–98% overlap of genets). Between the genic and random loci skim‐seq datasets, the overlap was 71%–95%, while the overlap between skim‐seq and GBS datasets were lower at 41%–82% range. Given the high level of overlap between selected genets regardless of genotypic dataset used, and the typical focus of plant breeding programs on multiple traits, breeding progress could be achieved under any method.

In an effort to understand the robustness of STITCH imputation impact on genomic prediction and how this may change based on filtering criteria, we developed several genotypic datasets using different numbers of markers and filtering methods to compare to GBS. Across five predicted traits, we found statistically significant difference in the cross‐validation using different marker set. However, in practice these differences are minimal and not consistently better for any given marker set (Table 2). Our results did not indicate a consistent advantage to any one method; for example, often 32,000 random markers performed as well as 96,000 random markers or GBS markers (Table 2). While there may have been an advantage to the gene space markers, it did not appear that there was a consistent trend or improvement by adding more than one locus per gene space. For shattering trait, markers from gene spaces performed better than random markers, although again in breeding application this superior performance would be minimal. Overall, STITCH imputation appeared to be quite robust, allowing us to develop simple, efficient pipelines that did not require substantial pipeline tuning and testing.

## 4 DISCUSSION

Genotyping via whole genome sequencing is attractive because of its broad coverage of markers across the genome and allows the greatest flexibility and future proofing of data for use and reuse in additional studies. However, obtaining high‐confidence genotypes has typically required sequencing at a higher depth (∼10x or more). Higher depth sequencing is particularly important when genotyping individuals that are polyploid or have high heterozygosity. Corresponding to this, high‐coverage sequencing becomes extremely cost prohibitive for a large genome species such as IWG. While sequencing costs have continued to decrease, it is still too costly to sequence large breeding populations at high enough coverage to obtain accurate genotype calls.

We tested a promising alternative of skim‐sequencing a complex, polyploid, cross‐pollinated highly heterozygous species at ultra‐low coverage by using an imputation algorithm specifically designed for low‐coverage sequencing data that leverages large population size to compensate for low sample‐level coverage. While skim‐seq has been proposed previously for genotyping and genomic analysis, here we focus on the methods to utilize low‐coverage data. Previous work has defined skim‐seq as 1x–2x coverage (Kumar et al. 2021) or been confined to very structured populations (Malmberg et al., 2018). Other skim‐seq has evaluated sequencing through long read sequencing, which may be useful in polyploid species (Malmberg et al., 2019). Even with a targeted coverage of 0.05x, we were able to obtain accurate genotype dosages for a high number of markers that can be utilized for GS. We sub‐sampled different numbers of imputed loci and compared GS prediction accuracy with our existing GBS pipeline. While we found statistically significant differences in correlations based on genotyping datasets, the difference between the highest and the lowest correlation was usually small and variable such that no one genotypic dataset was the best for every trait. In practice, we consider these correlations to be equivalent for plant breeding. Hence, the skim‐seq pipeline is a good substitute for existing GS pipelines using GBS (Crain et al., 2020; Crain, DeHaan, et al., 2021; Crain, Haghighattalab, et al., 2021) and SNP arrays (Elbasyoni et al., 2018; Poland, Brown, et al., 2012; Poland, Endelman, et al., 2012).

Imputation in STITCH requires specification of number of ancestral haplotypes, with smaller *K* giving faster imputation. Even with *K* = 4, we demonstrated a potential of obtaining sufficient density of loci for GS. For the studied population, *K* = 8 or *K* = 12 gave the best balance between speed and high number of loci for subsequent use.

Even modest info score thresholds above 0.40 resulted in genotype concordance above 0.95 and IQS > 0.85. However, *R* ^2^ approached 0.90 or higher only at info score thresholds above 0.80. Concordance and IQS are based only on high‐confidence imputed genotypes, while *R* ^2^ is based on all imputed data, explaining the comparatively higher values of concordance and IQS. In trying to optimize imputation parameters, we observed that STITCH is quite robust to a variety of settings (*K* and nGen), matching observations by Davies et al. (2021). This imputation robustness and ability to filter for higher confidence imputed loci (info score, concordance, *R* ^2^) should allow breeding programs to readily implement bioinformatic pipelines and successful GS even with unknown population parameters.

As a workaround for missing genotype calls in the STITCH output, Teng et al. (2022) suggested a two‐stage imputation method where high‐confidence genotype calls from STITCH are used as input for another imputation software such as Beagle to obtain a dataset with no missing genotype calls. However, when using imputed data for analysis that accept continuous quantitative data, it is recommended to simply use allelic dosage instead of discrete genotypes as it better represents the inherent uncertainty in imputation (Zheng et al., 2011). For developing the genomic relationship matrices, the quantitative dosage values were applicable to calculation of the relationship matrix in rrBLUP, similar to an imputation based on mean value for each marker (Endelman, 2011).

Sequencing more samples at lower coverage rather than fewer samples at higher coverage can improve the power and accuracy of inference for most genetic studies that benefit from sampling a larger population (Lou et al., 2021). The TLI and FRR breeding programs genotype a combined 10,000 samples annually for GS. In this paper, we attained good imputation accuracy for a high density of markers at an ultra‐low target coverage of 0.05x for nearly 10,000 samples. Similar to GBS, the implementation of skim‐seq and imputation with STITCH for a breeding program can proceed directly on large populations with low coverage, without the need for developing a specific panel with high‐coverage sequencing for variant discovery. Utilizing breeding populations suggests that enough individuals are available for sequencing; however, further work could evaluate the effect of sample size of individuals and number of markers. Within IWG, previous work (Crain, Haghighattalab, et al., 2021) showed the impact of varying sample size from 250 to 2000 individuals along with changing GBS marker numbers from 250 to 20,000. Based on current results, skim‐seq could likely be used at these minimal levels for breeding programs implementing GS. We utilized marker numbers in GS models that far exceeded our previous work in IWG (Crain, DeHaan, et al., 2021; Crain, Haghighattalab, et al., 2021), yet there is ample opportunity to expand the marker dataset through skim‐seq through more than 100,000 markers. The additional markers may prove useful in dissecting trait architecture through genome‐wide association studies.

In subsequent breeding cycles, we have used the skim‐seq pipeline described in this paper to impute nearly 20,000 samples for GS. These are among the lowest sequencing coverage and the largest sample sizes to be imputed with STITCH to date. Most studies have used STITCH for imputation of WGS data at around 1.0x to 3.0x coverage for 1000 to 2000 samples. The lowest sequencing coverage reported to be imputed with STITCH is 0.11x in humans (Xiao et al., 2024), 0.27x in rats (D. Chen et al., 2025), and 0.30x in inbred wheat lines (Clouard and Nettelblad, 2024). The most samples to have been imputed are 36,949 humans (Xiao et al., 2024) and 8760 rats (D. Chen et al., 2025). Given these results, STITCH appears to scale to plant breeding program levels and can be used across multiple species.

A key requirement for using this approach is a good‐quality reference genome. Fortunately, the development of reference genomes is increasingly being prioritized by different plant research communities, even for previously neglected and under‐researched species (Jamnadass et al., 2020). In the 20 years since the publication of the first plant reference genome for *Arabidopsis thaliana* in 2000, the plant science community has generated over 1000 reference genomes representing 788 plant species (Sun et al., 2022). Additionally, technological advances in long‐read sequencing have made the development of references genomes for any species much more tractable than ever before (Li et al., 2022). The skim‐seq pipeline presented is promising for application in plant breeding programs that have large populations. At scale, we estimate that sample DNA extraction, library preparation, and sequencing is approximately $10 USD per sample, excluding labor. This is more affordable than many other genotyping‐based options like GBS and arrays (Bassi et al., 2016). While skim‐seq currently requires access to computational resources and bioinformatics, advances in computing technology and increased utilization of skim‐seq will result in more user‐friendly pipelines (Glaubitz et al., 2014). The flexibility, scalability, and ease of application will make skim‐seq a popular system for GS and other population genetics studies, even for novel and under‐researched crops.

## AUTHOR CONTRIBUTIONS

**Sajal R. Sthapit**: Formal analysis; investigation; methodology; visualization; writing—original draft. **Jared Crain**: Data curation; formal analysis; investigation; methodology; writing—review and editing. **Steve Larson**: Investigation; resources; writing—review and editing. **James A. Anderson**: Funding acquisition; resources; writing—review and editing. **Prabin Bajgain**: Resources; writing—review and editing. **Lee R. DeHaan**: Conceptualization; investigation; resources; writing—review and editing. **Jesse Poland**: Conceptualization; methodology; writing—review and editing.

## CONFLICT OF INTEREST STATEMENT

The authors declare no conflicts of interest.

## Supporting information



Supplemental Materials

## Data Availability

The genomic data used and generated in this study have been deposited in NCBI Sequence Read Archive (SRA) (https://www.ncbi.nlm.nih.gov/bioproject/) as part of umbrella PRJNA609325. Data scripts to implement STITCH imputation and analysis of results has been uploaded to the Zenodo digital repository: https://doi.org/10.5281/zenodo.15611481.
