# Supplementary material for: A low‐coverage skim‐sequencing and imputation pipeline for genomic selection
Source: Plant Genome. 2025 Oct 23;18(4):e70139. doi: 10.1002/tpg2.70139 (PMC12547641; doi:10.1002/tpg2.70139)
Supplement: Supplementary file 1 [file TPG2-18-e70139-s001.docx]

# Supplemental Materials

Supplemental Figure S1. Relationship between number of variants (biallelic loci with minor allele frequency > 5%) ascertained from 445 samples and the size of the chromosome. Label colors red, green, and blue represent genomes J, S, and V respectively. Pearson correlation coefficient, r = 0.77 (df=19).

Supplemental Figure S2. Distribution of 28.2 million variants (y-axis) ascertained from whole-genome sequencing of 445 IWG samples at ~2x coverage along the length of the chromosome (x-axis) represented in 1 Mb bins. Dotted red lines are centromere location.

Supplemental Figure S3. Distribution of 31,393 GBS markers (y-axis) across the length of the chromosome (x-axis) presented in 1Mb bins. Dotted red lines are centromere location.

Supplemental Figure S4. Distribution of high-quality whole-genome sequencing markers within 5000bp of IWG gene models.

| (a)   | (b)   | (c)   |
| --- | --- | --- |

Supplemental Figure S5. Box and whisker plots of a) genotype concordance, b) imputation quality score (IQS), and c) R^2^ at 10 levels of info scores from STITCH imputation using K=8 and 0.04x down sampled coverage of 46 validation set samples. Red squares indicate mean values, and gray circles are outlier values.

Supplemental Figure S6. Mean concordance (P_o_), imputation quality score (IQS), and squared correlations (R^2^) between validation set genotype calls and imputed alternate allele dosages at 4 sequencing coverages (0.01x, 0.04x, 0.07x, and 0.10x) and 4 ancestral haplotypes (4, 8, 12, 16, 20, 24, and 28) for 21 IWG chromosomes (1J to 7V). Imputed loci were filtered at info score > 0.80.

Supplemental Figure S7. Correlation of predicted values for multiple phenotypes, a) plant height, b) seed mass, c) seed shattering, and d) spike yield obtained from using different genotyping datasets. GBS, genotyping-by-sequencing; lpg, locus per gene.

Supplemental Figure S8. Proportion of samples overlapping among the lowest predicted phenotypes values of a) plant height, b) seed mass, c) seed shattering, and d) spike yield obtained from using different genotyping datasets. Note: gbs, genotyping-by-sequencing; lpg, locus per gene.

Supplemental Figure S9. Proportion of samples overlapping among the highest predicted phenotypes values of a) plant height, b) seed mass, c) seed shattering, and d) spike yield obtained from using different genotyping datasets. Note: gbs, genotyping-by-sequencing; lpg, locus per gene.

Supplemental Table S1. Variants (loci with two alleles and minor allele frequency > 5%) ascertained from whole-genome sequencing (WGS) of intermediate wheatgrass samples.

| **Chrom** | **Base Pairs** | **Number of Genes** | **Variants from 445 samples at ~2x coverage** | **Variants from 46 samples at ~17x coverage** |
| --- | --- | --- | --- | --- |
| 1J | 548,726,614 | 5940 | 1578865 | 5040778 |
| 2J | 694,191,331 | 7637 | 1907790 | 7417442 |
| 3J | 664,523,050 | 6895 | 1889667 | 6725106 |
| 4J | 541,250,370 | 4767 | 1458942 | 4637394 |
| 5J | 602,542,912 | 6966 | 1571616 | 5229698 |
| 6J | 566,711,382 | 5825 | 1492553 | 4953222 |
| 7J | 686,173,349 | 7383 | 1959282 | 6640801 |
| 1S | 422,989,602 | 5556 | 996442 | 3361645 |
| 2S | 507,709,987 | 7200 | 1089408 | 3619900 |
| 3S | 493,345,210 | 6781 | 1178576 | 3635982 |
| 4S | 377,353,484 | 4718 | 1362226 | 4232810 |
| 5S | 549,394,082 | 7369 | 1415357 | 4575561 |
| 6S | 408,503,727 | 5412 | 872828 | 3151804 |
| 7S | 482,783,349 | 6765 | 1079426 | 3276328 |
| 1V | 574,115,619 | 5657 | 1025311 | 4039119 |
| 2V | 618,570,211 | 7032 | 1196715 | 4747717 |
| 3V | 526,408,016 | 6238 | 900053 | 3994625 |
| 4V | 490,142,664 | 4633 | 1114819 | 3310703 |
| 5V | 591,402,192 | 6277 | 1485452 | 5137545 |
| 6V | 462,045,100 | 5259 | 880202 | 3141209 |
| 7V | 761,377,749 | 6649 | 1773654 | 6382488 |
| **Total** | **11,570,260,000** | **130,959** | **28229184** | **97251877** |
| **Mean** | **550,964,762** | **6236** | **1344247** | **4631042** |
